# Resveratrol reverses the adverse effects of bevacizumab on cultured ARPE-19 cells

**DOI:** 10.1038/s41598-017-12496-z

**Published:** 2017-09-25

**Authors:** Murali Subramani, Murugeswari Ponnalagu, Lekshmi Krishna, Nallathambi Jeyabalan, Priyanka Chevour, Anupam Sharma, Chaitra Jayadev, Rohit Shetty, Nargis Begum, Govindaraju Archunan, Debashish Das

**Affiliations:** 1 Stem Cell Lab, GROW Laboratories, Narayana Nethralaya Foundation, Bangalore, Karnataka India; 2 GROW Laboratories, Narayana Nethralaya Foundation, Bangalore, Karnataka India; 3 Vitreoretina Services, Narayana Nethralaya Eye Hospital, Bangalore, Karnataka India; 4 Department of Cornea and Refractive Surgery, Narayana Nethralaya Eye Hospital, Bangalore, Karnataka India; 5 Postgraduate Department of Biotechnology, Jamal Mohammed College, Tiruchirappalli, Tamilnadu India; 6 Department of Animal Science, Bharatidasan University, Tiruchirappalli, Tamilnadu India

## Abstract

Age-related macular degeneration (AMD) and proliferative diabetic retinopathy (PDR) are one of the major causes of blindness caused by neo-vascular changes in the retina. Intravitreal anti-VEGF injections are widely used in the treatment of wet-AMD and PDR. A significant percentage of treated patients have complications of repeated injections. Resveratrol (RES) is a polyphenol phytoalexin with anti-oxidative, anti-inflammatory and anti-proliferative properties. Hence, we hypothesized that if RES is used in combination with bevacizumab (BEV, anti-VEGF), it could reverse the adverse effects that precipitate fibrotic changes, drusen formation, tractional retinal detachment and so on. Human retinal pigment epithelial cells were treated with various combinations of BEV and RES. There was partial reduction in secreted VEGF levels compared to untreated controls. Epithelial-mesenchymal transition was lower in BEV + RES treated cultures compared to BEV treated cultures. The proliferation status was similar in BEV + RES as well as BEV treated cultures both groups. Phagocytosis was enhanced in the presence of BEV + RES compared to BEV. Furthermore, we observed that notch signaling was involved in reversing the adverse effects of BEV. This study paves way for a combinatorial strategy to treat as well as prevent adverse effects of therapy in patients with wet AMD and PDR.

## Introduction

In vasoproliferative ocular diseases such proliferative diabetic retinopathy (PDR), retinal vein occlusion (RVO), and wet-age related macular degeneration (AMD), an important therapeutic target is vascular endothelial growth factor (VEGF) in the form of intravitreal injections of anti-VEGF agents^1,2^. Most often there is a need for multiple injections to ensure adequate regression of the disease and to counter recurrences^3,4^. Despite the potential risks of repeated injections of anti-VEGF over prolonged periods of time, the lack of an alternative makes it the most widely used treatment regime for neo-vascular retinal diseases.

Among the anti-VEGF agents, the most widely used in clinical practice are bevacizumab (BEV, Avastin^®^, Genentech/Roche, San Francisco, USA) followed by ranibizumab (RAN, Lucentis^®^, Novartis Pharma Stein AG, Switzerland)^5–7^. The popularity of the usage of BEV over RAN is primarily driven by the fact that though clinically they have similar functions, the BEV is much affordable than RAN and hence popular in developing nations^6^. The retinal pigment epithelial (RPE) cell layer, that is adjacent to the photoreceptor layer, is a key cellular layer in ocular neo-vascular diseases as the pro-angiogenic factor VEGF is predominantly secreted here^8,9^. Hence, it remains a key site of action for all the anti-VEGF treatments. *In vitro* as well as animal model experiments have demonstrated several adverse effects of long term and short term exposure of BEV therapy^10–12^. *In vitro* studies have shown that BEV gets internalized into the cultured RPE cells^13^. This intracellular accumulation of BEV results in reduced phagocytic property of these cells and also affects the RPE barrier function^14,15^. Moreover, intracellular accumulation of anti-VEGF agents has been shown to reduce intracellular VEGF-A levels, thereby affecting its metabolism^16^.

Clinical dosage of BEV has been shown to mildly reduce proliferation, and with a higher concentration or with high glucose levels, it caused cytotoxicity in cultured RPE cells^17–19^. Clinical dosage of BEV upregulates CTGF leading to pro-fibrotic changes with increased loss of epithelial properties in cultured RPE cells resulting in induction of epithelial-mesenchymal transition (EMT)^20^. We have previously shown that a short exposure of clinical concentration of BEV in cultured human RPE cells reduces cell proliferation and phagocytosis with increased epithelial-mesenchymal transition (EMT) and transmembrane potential^7^. Results from animal and clinical studies have revealed the most complications of BEV treatment are vitreous hemorrhage, tractional retinal detachment, fibrotic membrane formation and retinal pigment epithelial tears^21,22,7,10^. There are also reports on macular atrophy occurring after repeated injections of anti-VEGF for wet AMD^23^. Clinical trials like ANCHOR, MARINA and CATT study have reported that 8–10% of patients on treatment with anti-VEGF agents develop dry AMD like phenotype with geographic atrophy^24–27^. Moreover, despite adequate treatment, there remains a cohort of ~40% and ~45% anti-VEGF non-responders with PDR and AMD respectively^28,29^. The above factors necessitate the need for alternatives as well as combinatorial therapy without compromising treatment efficacy.

We investigated the influence of RES, a stilbenoid natural polyphenol phytoalexin, as a potential protective agent. It is found in the skin of grapes, berries and peanuts and exerts its anti-oxidant, anti-inflammatory, anti-epithelial-mesenchymal transition and anti-proliferative roles through sirtuin 1^30,31^. RES has been used in the treatment of diabetic retinopathy and dry AMD due to its anti-angiogenic and enhanced phagocytic properties, respectively^32^. In a cell culture model RES inhibited EMT induced by TGF-β, thereby restoring the ZO-1 and α-SMA staining and reducing the expression of mesenchymal marker vimentin by suppressing Smad2 and Smad3 phosphorylation^33^. Studies have shown that impaired autophagy, a major driver for AMD can be restored in the presence of Resvega suggesting a plausible therapeutic window for treating AMD^34^. By regulating PCNA, p21, p27 and p38MAPK/MMP-9 expression, RES can block proliferation and migration in ARPE-19 cells^35^. Administration of dietary RES reduces inflammatory, senescence and oxidative stress markers in trabecular meshwork cells exposed to 40% O2^36^. Retinal ganglion cell death has been shown to be prevented by a dietary supplement of RES in an optic nerve crush mice model^37–39^. It has also been shown that RES, through activation of SIRT1, downregulated IL-17 in mononuclear cultures of PVR patients suggesting a protective mechanism of RES in DR progression^40^. Oxidative stress induced lens epithelial cultures were protected from apoptosis by SIRT1 inhibition of p53 pathway^41^. In cultured RPE cells, RES caused induction of mesenchymal-epithelial transition besides blocking TGF-β induced EMT, proliferation and migration by activation of SIRT1^31^. RES reduces the inflammatory response induced by endotoxins in uveitis and is effective in controlling retinopathy of prematurity by regulating nitric oxide levels in animal models^30,42^. RES can restore RPE phagocytosis property in UV-exposed RPE cultures through ion channels^43^. Apart from the activation of SIRT1, RES acts through the Smad2, Smad4 and PI3K/Akt/mTOR pathways^44^. RES has also been shown to activate Notch signaling in both ocular and non-ocular cells^45,46^.

Notch signaling is a developmentally regulated, conserved signaling pathway involved in angiogenesis and VEGF regulation, apart from its role in cell fate decision, cell-to-cell communication, proliferation and apoptosis. There are several studies implicating cross-talk between notch signaling and VEGF^47^. The stringency in the culturing conditions of RPE also has been shown to be decisive with respect to the expression of Notch signaling. We have shown that RPE cells cultured on de-epithelialized amniotic membrane down regulated Notch signaling when compared to controls^8^. We have previously depicted that a short pulse of BEV is sufficient enough to dampen Notch signaling activity, which plays an important role in epithelial-mesenchymal transition, proliferation, membrane potential and phagocytic properties of RPE^7^. Members of Notch signaling pathway, Notch 1, 4 and Delta like 4 play crucial roles during vasculogenesis and angiogenesis^48,49^.

In order to exploit the protective potential of RES, we investigated the response of human RPE cultures to RES and BEV alone and in combination. It’s role in mitigating the adverse effects of anti-VEGF agents and on other properties of RPE cells such as secretion of VEGF, phagocytosis, proliferation, apoptosis, EMT induction and membrane potential have been studied.

## Results

### VEGF expression and secretion patterns in RES and/or BEV treated ARPE-19 cells

**Figure-1:**
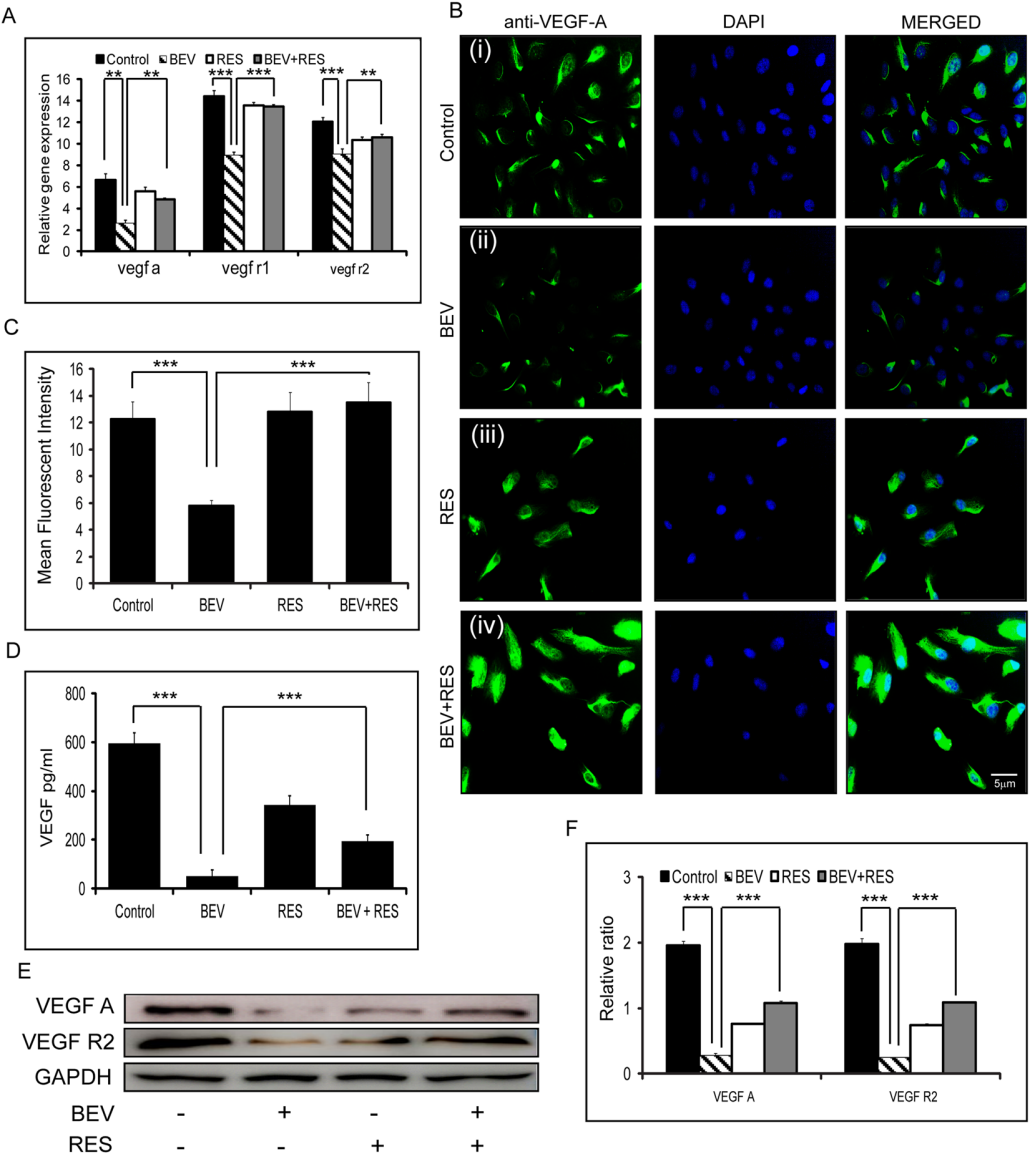
Effects of BEV and/or RES on VEGF and its receptors. Bar graph shows the relative gene expressions of vegf and its receptors r1 and r2 with respect to mRNA levels of gapdh in ARPE-19 cells treated with BEV, RES and BEV + RES (**A**). Representative immunofluorescent images showing the VEGF (green) and DAPI (blue) staining in ARPE-19 cells after various treatments (**B**). Mean fluorescent intensity measured using Image J software and represented graphically (**C**). Graphical representation of the secreted VEGF concentration was measured using sandwich ELISA (**D**). Representative western blot analysis for VEGF, VEGF-R2 and GAPDH in ARPE-19 cells incubated with BEV, RES, BEV + RES and untreated cultures (**E**). Graphical representation of the quantified western blot analysis with respect to GAPDH protein expression (**F**). Statistical analysis was performed using student’s t-test (**P < 0.01, P*** < 0.005) (n ≥ 3). Scale bar = 5 µm.

Gene expression of vegf a (p = 0.007), vegf r1 (p = 0.003) and vegf r2 (p = 0.002) was significantly decreased in cells treated with BEV when compared to untreated controls. There was no significant difference in the mRNA levels of vegf a, vegf r1 and vegf r2 between RES, BEV + RES and control groups. There was a significant difference in the mRNA levels of vegf a (p = 0.008), vegf r1 (p = 0.002), vegf r2 (p = 0.008) in BEV + RES treated cells when compared to BEV alone (Fig. 1A). We further looked at the cellular regulation of VEGF A, VEGF R1 and VEGF R2 in human RPE cells treated with BEV and RES alone or in combination. The results of the mean fluorescent intensity revealed a significant decrease in the levels of VEGF immunofluorescence staining in cells treated with BEV in comparison to untreated controls (p = 0.0002) and BEV + RES (p = 0.0002) treated cells (Fig. 1B and C). Apart from the cellular VEGF levels, we looked at the secreted VEGF form in the cell culture supernatant. While controls showed the highest levels of VEGF compared to the treated cells. BEV neutralized the secreted VEGF whereas RES and RES + BEV treated cells partially neutralized the secreted VEGF levels. Secreted VEGF levels were significantly higher (p = 0.0004) in BEV + RES treated cultures compared to BEV alone treated cultures (Fig. 1D). Western blot analysis revealed results similar to that of immunofluorescence staining. BEV treated cells showed significantly lower amounts of VEGF A and VEGF R2 than the untreated controls (p = 0.0003; p = 0.002) and BEV + RES (p = 0.002; p = 0.002) treated cells, respectively (Fig. 1E and F).

### Epithelial-mesenchymal transition (EMT) is influenced by BEV and RES

**Figure-2:**
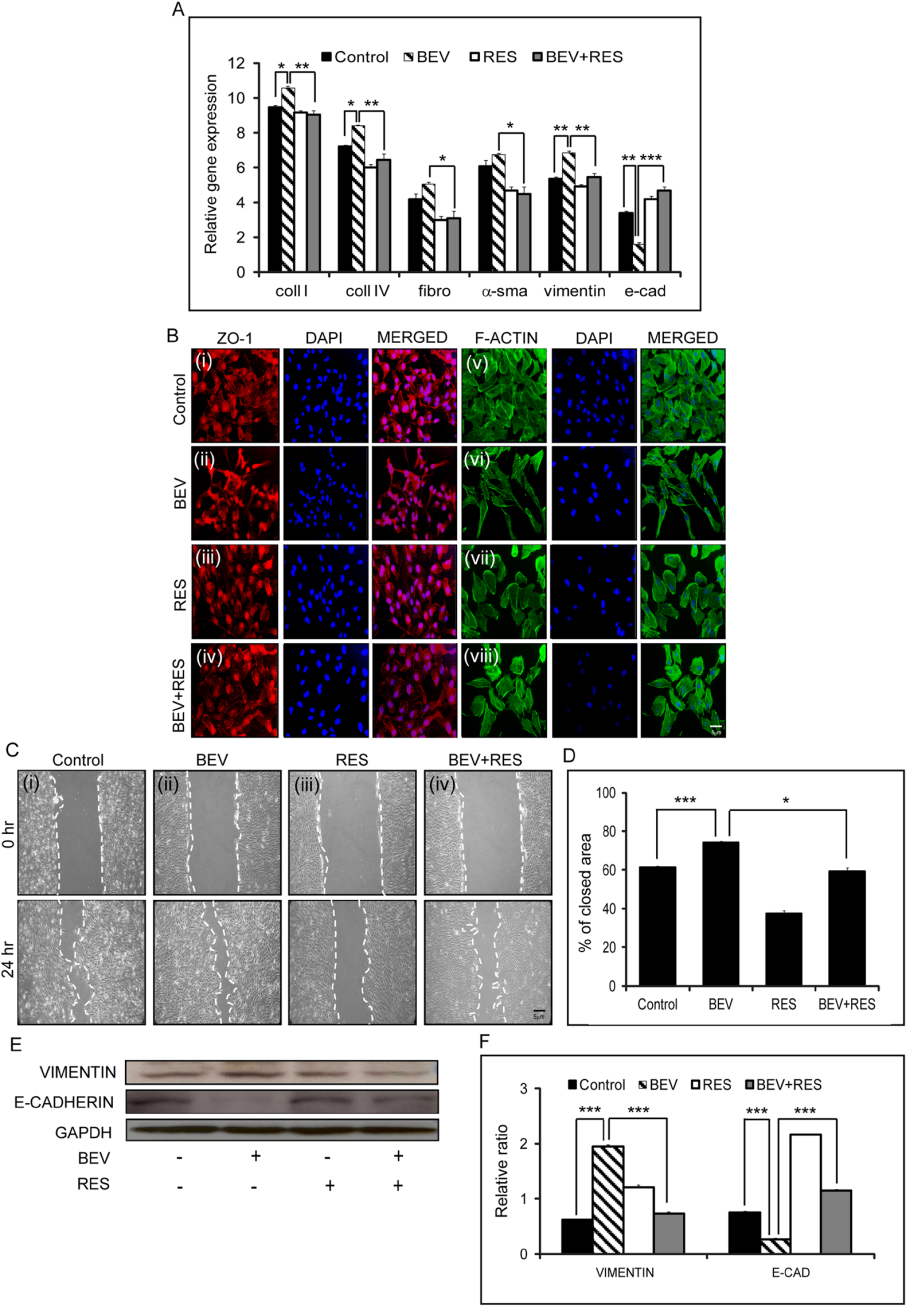
Effects of BEV + RES treatment on Epithelial-Mesenchymal transition of ARPE-19 cells. Quantitative PCR results of ARPE-19 cells treated with BEV, RES, BEV + RES and untreated controls. Graphical representation of the relative mRNA levels of collagen 1 (coll 1), collagen 4 (coll 4), Fibronectin (fibro), α-smooth muscle actin (α-sma), vimentin and e-cadherin (e-cad) with respect to mRNA levels of gapdh (**A**). Representative immunofluorescence staining of ZO-1 (red) (i−iv), F-ACTIN phalloidin (green) (v−viii) and DAPI (blue) in cultured ARPE-19 cells with untreated controls, and treated with BEV, RES and BEV + RES. (**B**) Representative phase contrast microscopic images showing the scratch assay in untreated ARPE-19 cultures along with cultures treated with BEV, RES and BEV + RES at two different time points (0 hr and 24 hrs) (**C**). Graphical representation of the quantified closed area depicted as percentage of close area in scratch assay (**D**). Representative western blot analysis for the EMT markers VIMENTIN and E- CADHERIN in ARPE-19 cells exposed to BEV, RES and BEV + RES and untreated controls (**E**). Graphical representation of densitometric analysis of western blot quantification represented with respect to the expression levels of GAPDH (**F**). Statistical analysis was performed using student’s t-test (*P < 0.05,**P < 0.01, ***P < 0.005) (n ≥ 3). Scale bar = 5 µm.

Expression of EMT genes was studied in untreated and treated (BEV, RES, BEV + RES) cells Epithelial markers (e-cadherin), mesenchymal markers (vimentin, fibronectin, a-smooth muscle actin) and extracellular matrix markers (collagen I and collagen IV) were used to investigate the effect of BEV and RES on EMT.Gene expression profile of coll I, coll IV and vimentin were significantly increased in BEV treated cells in comparison to untreated controls (p ≤ 0.05), RES (p ≤ 0.05) and BEV + RES (p ≤ 0.05) treated cells. Fibronectin and α-smooth muscle actin (α−sma) mRNA showed elevated expression though it was not significant in comparison to controls. On the contrary, e-cadherin expression was significantly low in cells treated with BEV compared to controls (p = 0.007) and BEV + RES (p = 0.003) treated cells (Fig. 2A). Immunofluorescence staining with ZO-1 and F-ACTIN showed a well demarcated cellular boundary in controls, RES and BEV + RES treated cells but was absent in BEV treated cells. There was a marked appearance of the cell margin staining by ZO-1 in cells treated with BEV + RES which was not present in the BEV treated cells (Fig. 2B i–iv). Actin cytoskeleton status was evaluated with Alexa Fluor 488 phalloidin staining. Controls as well as RES treated cells revealed lesser stress fibers compared to BEV treated cells. The cell shape was more elongated and mesenchymal-like in BEV treated cells. The cellular morphology of BEV + RES treated cells was similar to control s (Fig. 2B v–viii). Scratch assay was performed to revalidate the EMT status of the treated cells. A 61.2% closure of the cells in the control group was noted that was significantly lower (p = 0.0005) than the closure in BEV (74.2%) treated cells. Cells treated with BEV + RES showed 59.4% closure that was similar to that of untreated controls. There was a significant upregulation in the percentage of closure in BEV treated cells compared to controls (p = 0.0005) and BEV + RES (p = 0.015) treated cells (Fig. 2C and D). Western blot densitometric analysis revealed a significant upregulation in the expression of VIMENTIN and a concurrent significant reduction in the levels of E-CADHERIN in BEV treated cells in comparison to controls (p = 0.0001; p = 0.0002) and BEV + RES (p ≤ 0.005) treated cells (Fig. 2E and F).

### Effects of BEV and RES on cell proliferation

**Figure-3:**
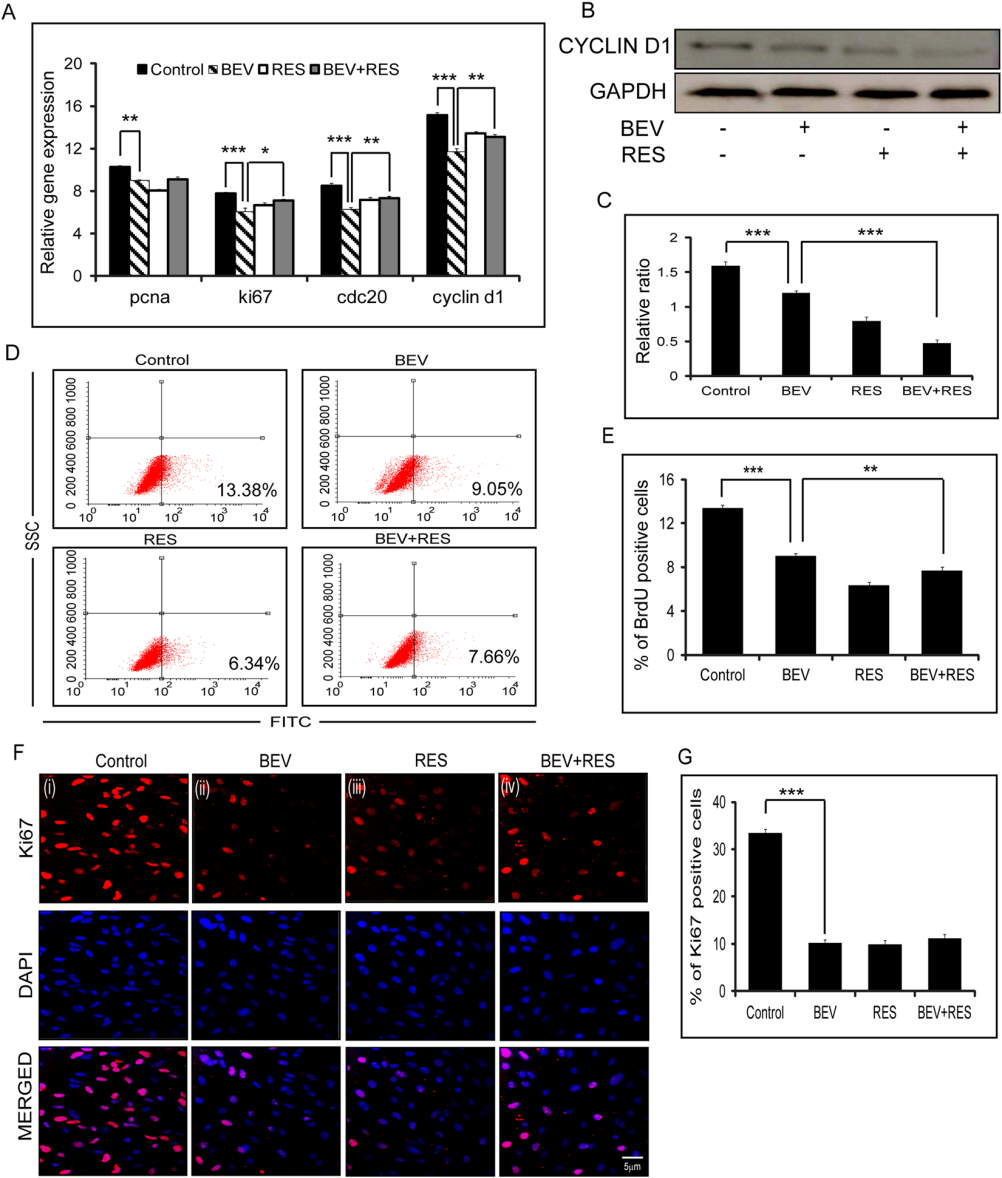
Cell proliferation status of ARPE-19 cells with BEV, RES and BEV + RES treatments. Graphical representation of the levels of mRNA expression of pcna, ki67, cdc20 and cyclin d1 with respect to mRNA levels of gapdh in ARPE-19 cells treated with BEV, RES, BEV + RES and untreated controls as estimated by quantitative real-time PCR. The mRNA levels of gapdh were taken as control (**A**). Representative western blot results of CYCLIN D1 in untreated and treated ARPE-19 cells (**B**). Densitometric analysis of the expression of CYCLIN D1 with respect to the GAPDH levels is plotted graphically (**C**). Representative FACS plots depicting the BrdU positivity in treated and untreated ARPE-19 cells as shown in percentage of positivity (**D**). Graphical representation of percentage of BrdU positivity in untreated and cells treated with BEV, RES and BEV + RES (**E**). Representative immunofluorescence images of Ki-67 positivity (red) and DAPI (blue) in untreated cells and cells treated with BEV, RES and BEV + RES (**F**). Percentage of Ki-67 positivity is represented in bar graphs by counting the number of merged (red and blue) cells (**G**). Statistical analysis was performed using student’s t-test (*P < 0.05,**P < 0.01, P*** < 0.005) (n ≥ 3). Scale bar = 5 µm.

Cell proliferation was assayed in cells after treatment with BEV, RES and BEV + RES. A brief exposure to BEV resulted in a significant decrease in the expression of pcna (p = 0.007), ki67 (p = 0.002), cdc20 (p = 0.0006) and cyclin d1 (p = 0.003) in comparison to controls. There was a significant difference in the mRNA levels of Ki67 (p = 0.025), Cdc20 (p = 0.006) and Cyclin D1 (p = 0.006) in cells treated with BEV in comparison to cells treated with BEV + RES (Fig. 3A). Western blot analysis revealed that BEV treated cells had a significantly (p = 0.0003) decreased CYCLIN D1 protein expression compared to controls. In the presence of BEV + RES, the protein level of CYCLIN D1 further decreased (p = 0.0001) in comparison to cells treated with BEV alone (Fig. 3B and C). BrdU labeling analysis showed a significant decrease in BrdU positivity in cells treated with BEV in comparison to controls (p = 0.0003). A significant decrease was observed in cells treated with BEV + RES in comparison to those treated with BEV (p = 0.005) alone (Fig. 3D and E). Furthermore, immunofluorescence staining with Ki67 showed a significant decrease in nuclear staining positivity in cells treated with BEV in comparison to untreated (p = 0.003) cells. There was no significant difference in the percentage of Ki67 positivity between BEV treated compared to BEV + RES treated cells (Fig. 3F,G).

### RES and BEV affect RPE phagocytosis

**Figure-4:**
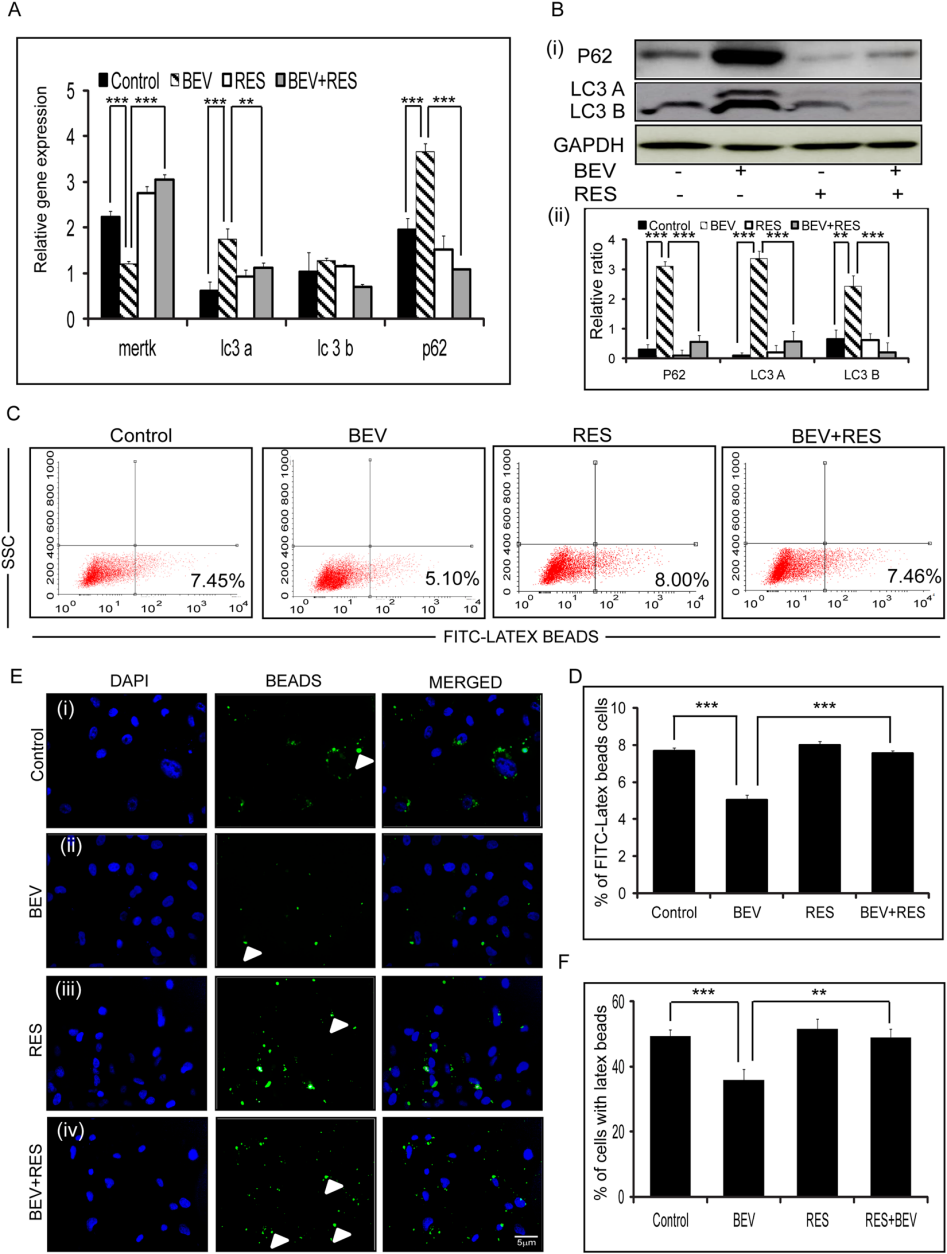
BEV + RES influences on phagocytosis activity. Graphical representation of the quantified real time PCR result showing the relative mRNA expression levels of mertk, lc3a, lc3b and p62 with respect to gapdh in untreated and cells treated with BEV, RES and BEV + RES (**A**). Representative Western blot images of p62, LC3A, LC3B and GAPDH in cells untreated or treated with BEV, RES and BEV + RES (**B**) (i). Graphical representation of densitometry based quantification of the Western blot (**B**) (ii). Representative images of FACS analysis depicting the percentage of cells with FITC-latex beads (**C**). Graphical representation of the FACS results showing the percentage of cells with FITC positivity (**D**). Representative immunofluorescence images of opsonized FITC-tagged latex beads (green) and nucleus stained (DAPI-Blue) images of untreated and treated ARPE-19 cells (**E**). Percentage of cells with latex beads were counted and have depicted graphically (**F**). Statistical analysis was performed using student’s t-test (***P < 0.005) (n ≥ 3). Scale bar = 5 µm.

A short exposure of BEV resulted in significantly reduced expressions of mertk (p = 0.0007; p = 0.0004), whereas a significant upregulation was noted in mRNA levels of lc3a (p = 0.002; p = 0.006), and p62 (p = 0.0003; p = 0.0001) mRNA compared to controls and RES + BEV treated cells, respectively. The reduction in the mRNA expression of mertk in the presence of BEV was restored partially in the presence of BEV + RES. However, there was no difference in the mRNA levels of lc3b in controls compared to RES + BEV treated cells (Fig. 4A). ARPE-19 cells treated with BEV, showed increase in the LC3-I, LC3-II and p62 levels compared to untreated or controls in Western blot. Contrarily, cells treated with BEV + RES showed decreased levels of LC3-I, LC3-II and p62 (Fig. 4B). RPE phagocytosis was studied using FITC labeled latex-beads in ARPE-19 cells treated with BEV, RES and BEV + RES. Phagocytosis assay was performed by quantifying the number of FITC tagged opsonized beads using flow cytometry. In BEV treated cells (5.1%) the percentage of opsonized beads was significantly lower than untreated control (7.45%, p ≤ 0.005) as well as BEV + RES (7.46%) treated cells (p ≤ 0.005, Fig. 4C and D). Immunofluorescence assay for phagocytosis was performed by counting the number of cells with opsonized beads. The results revealed a significant increase in the number of cells with beads in BEV + RES (p = 0.0002) treated cells compared to BEV treated cells. The number of cells with opsonized beads were also significantly low in BEV treated cells compared to controls (p ≤ 0.005, Fig. 4E and F).

### Effects of BEV and RES on membrane potential

**Figure-5:**
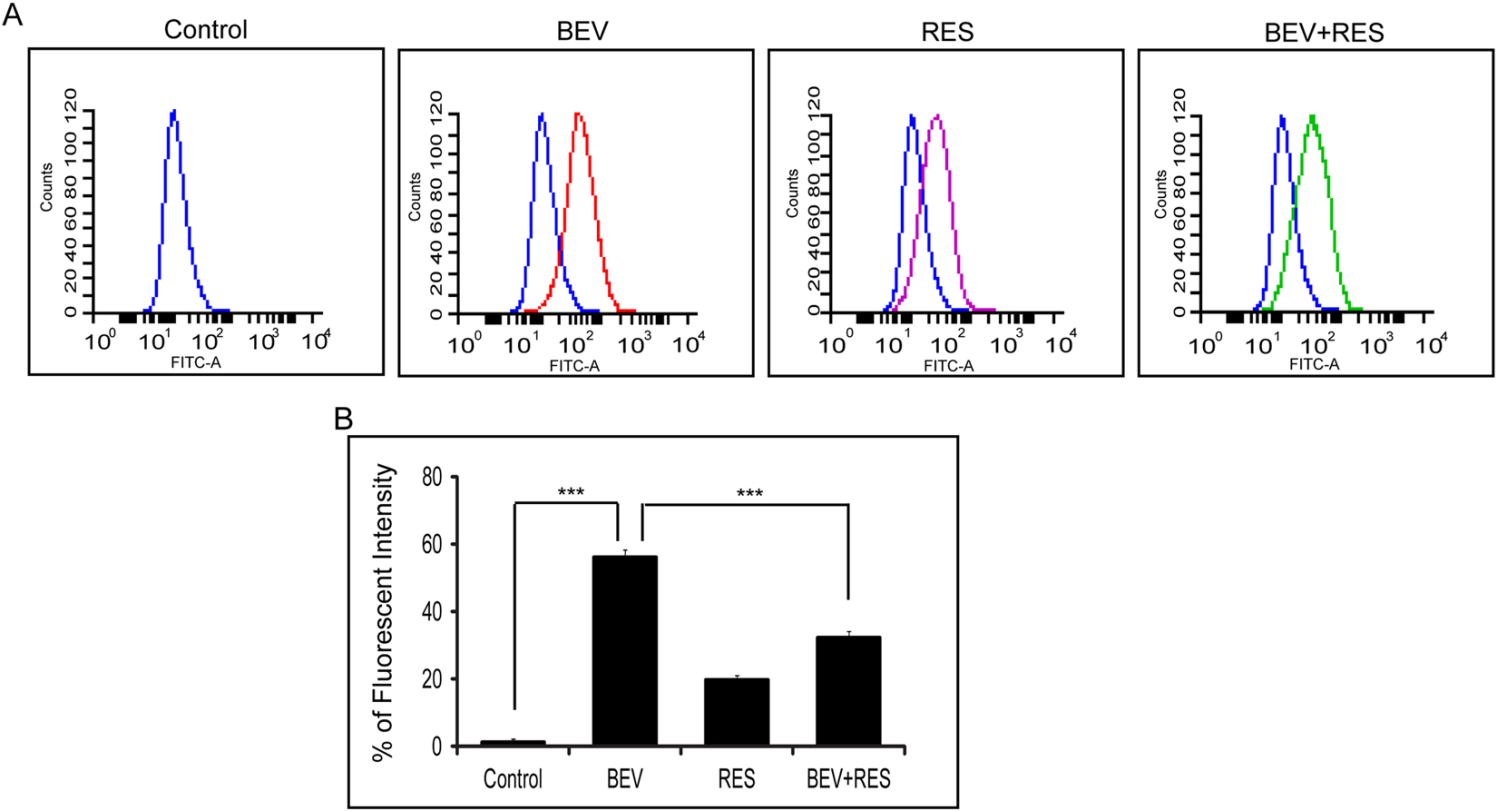
Effects of BEV + RES on transmembrane potential. DiBAC4 (3) dye was used to analyse the membrane potential of BEV,RES and BEV + RES treated cells. (**A**,**B**) The histogram depicts the shift in the peaks due do the internalized dye on various treatments. For each treatment the histogram peaks are represented with specific colours (**A**) Bar graph shows the mean fluorescent dye intake in the treated and control cells (**B**). Statistical analysis was performed using student’s t-test (P*** < 0.005) (n ≥ 3).

Since the membrane potential status of RPE is crucial for its proper functionality we looked at the effect of the BEV, RES and BEV + RES on cell polarization. Cultures exposed to BEV alone resulted in depolarization of the cell membrane and to RES alone caused cell membrane hyperpolarization. A combination of BEV + RES resulted in a significant decrease in the depolarization status of cells compared to BEV treated cells (p = 0.0003). BEV treated cells showed a higher depolarization in comparison to controls (p ≤ 0.005, Fig. 5A and B).

### Effect of BEV and RES on Notch 4 and Dll 4 signaling

**Figure-6:**
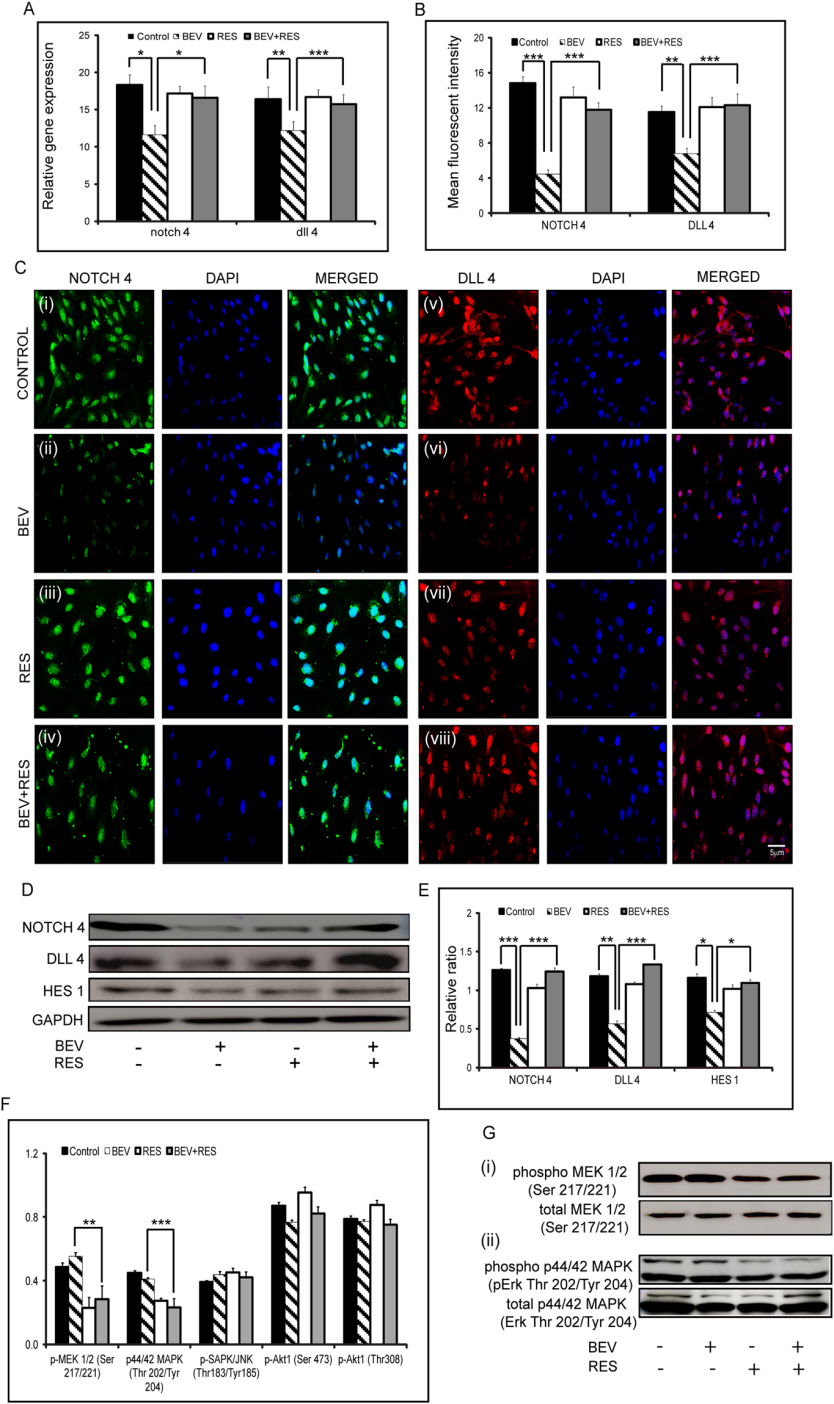
Effects of BEV + RES on Notch signaling. Bar graph depicts the relative mRNA expression levels of notch 4 and dll 4 in with respect to mRNA levels of gapdh in untreated ARPE-19 cells and those treated with BEV, RES and BEV + RES (**A**). Graphical representation of quantified mean fluorescent intensity of immunofluorescent images for NOTCH 4 and DLL 4 are shown (**B**). Representative immunofluorscent images showing positivity for NOTCH 4 and DLL4 in untreated and BEV, RES, BEV + RES treated ARPE-19 cells (**C**). Representative western blots of NOTCH 4, DLL4 and the downstream target HES 1 in untreated and treated ARPE-19 cells (**D**). Graphical representation of the densitometric analysis of western blot results with respect to the levels of GAPDH is shown (**E**). Graphical representation of PathScan^®^ ELISA results of untreated and cells treated with BEV, RES and BEV + RES (**F**). Representative Western blot showing phosphorylated and total MEK1/2 (i) and p44/42 MAPK (ii) (**G**). Statistical analysis was performed using student’s t-test (*P < 0.05,**P < 0.01, ***P < 0.005) (n ≥ 3). Scale bar = 5 µm.

We have previously shown that BEV exposed RPE cells show lower expression and signaling that is mediated by notch 4 and dll 4^7^. Gene expression of notch 4 and dll 4 were significantly lower in cells treated with BEV in comparison to controls (p = 0.03; p = 0.009) and cells treated with BEV + RES (p = 0.02; p = 0.003) (Fig. 6A). Immunofluorescence staining for NOTCH 4 and DLL4 showed that the mean fluorescent intensity of cells treated with BEV were significantly lower than those with BEV + RES (p = 0.001; p = 0.0005) and controls (p = 0.0008; p = 0.007). Additionally, there was no detectable difference in the mean fluorescent intensity in NOTCH 4 and DLL 4 levels in controls when compared to RES and BEV + RES treated cells (Fig. 6B and C). Furthermore, western blot analysis depicted a significantly lower expression of NOTCH 4 (p = 0.004) and DLL 4 (p = 0.002) in cells exposed to BEV in comparison to cells treated with BEV + RES (Fig. 6D and E). In an attempt to look at the status of downstream target of Notch signaling, the cells (treated and untreated) were immunoblotted for levels of HES1. Cells treated with BEV + RES showed significantly (p = 0.02) higher levels of HES1 in comparison to BEV alone treated cells (Fig. 6D and E). There was no significant difference in the levels of NOTCH 4, DLL4 and HES 1 in controls compared to cells treated with RES and BEV + RES.

In order to understand the plausible signaling pathway involved, we investigated the phosphorylation status of kinase pathway in absence and presence of RES, BEV and RES + BEV. PathScan® ELISA was performed for phosphor proteins, p-MEK1/2, p44/42 MAPK (Thr202/Tyr204), pSAPK/JNK (Thr183/Tyr185), pAkt (Ser473) and pAkt (Thr308). ELISA results revealed that there was significant decrease in the phosphorylation status in of pMEK1/2(Ser217/221) and p44/42 MAPK (Thr202/Tyr204) (Fig. 6F). The ELISA results were validated using Western blot for phosphorylated MEK1/2 and phosphorylated p44/42 MAPK (Thr202/Tyr 204) which showed decreased levels of phosphor MEK1/2 in ARPE-19 cells incubated with RES and RES + BEV compared to cells with BEV alone (Fig. 6G(i)). Similar results were also observed on phosphorylated p44/42 MAPK. In the presence of RES and BEV + RES there is decrease in the phosphorylated levels of p44/42 MAPK in comparison to that of BEV treated cells (Fig. 6G(ii)). These findings suggest that the restoration effects of RES treatment in ARPE cells is probably an outcome of the decreased phosphorylation levels of MEK1/2 and p42/44 MAPK.

## Discussion

Intravitreal injections of anti-VEGF agents is the most preferred therapeutic intervention for wet AMD, RVO and PDR, despite some severe adverse effects and need for repeated injections^5^. It remains popular due the lack of alternatives to address pathological neo-vascularization. One of the major limitations of anti-VEGF therapy is that it neutralizes all the available secreted VEGF, which is largely a homeostatic function of the RPE and its secretome^1,9^. This leads to deleterious effects on the vascular health of the retina as VEGF is essential for maintaining a viable retinal vascular architecture^10,50,51^.

Anti-VEGF agents such as BEV areknown to induce altered phagocytosis and enhance EMT in RPE cells both on short-term and long-term exposure^7,52^. BEV exposure in RPE cells has been shown to induce pro-fibrotic changes via EMT induction^20^. Clinical trials using intravitreal anti-VEGF injections have shown adverse effects in wet-AMD patients^27^. The loss of visual acuity was associated with changes in the RPE akin to geographic atrophy^53^. Hence, it is important to investigate combinatorial treatment strategies to prevent such complications. In such an effort, we compared the effect of anti-VEGF (BEV) with and without RES.

RES, a poly phenol found in red wine, has been shown to restore normal function in dry AMD in animal and cell culture studies^54^. RES has also been approved by the US Food and Drug Administration (FDA) as a dietary supplement. It is widely used in ophthalmology both for clinical as well as experimental studies using animal models and *in vitro* culture systems. Though RES reduces VEGF levels, it does not have the same efficacy as anti-VEGFs. It also maintains phagocytic properties in cultured RPE cells in adverse treatment conditions^55^. BEV reduces VEGF levels but also affect the normal cellular physiology of RPE cells^7^. RES has also been shown to have anti-oxidant, anti-inflammatory, anti-aging, anti-angiogenic properties^32,42^. Most of its functions are driven by sirtuin 1(SIRT1), that deacetylates transcription factors and other proteins^56^. Moreover, Resvega a dietary supplement containing resveratrol in combination with vitamins is developed based on the findings of Age-related eye disease study (AREDS), reduced the probability of developing dry AMD^57^. On the other aspect, most of the adverse effects of BEV treatment has been attributed to the intracellular accumulation of BEV^14^. It could be hypothesized that such an accumulation of BEV might induce oxidative stress to the cell machinery thereby leading to defective physiological functions of the RPE. The anti-oxidant role of RES has been well demonstrated in several studies^32,42,54^. With this background, we thus investigated the possible role of RES in reversing the adverse effects of anti-VEGF (BEV), thereby providing an option for a combined therapy of anti-VEGF and RES in wet AMD and PDR patients. Such a combinatorial therapy may prevent geographic atrophy and other complications such as RPE tear, fibrosis, drusen formation and so on.

The IC50 of RES differs for various cell lines^58,59^. In choroidal endothelial cells, IC50 for RES was determined to be 26 µM at the 96 hr time point^60^. In our study with ARPE-19 cells, IC50 for RES was calculated to be 625.3 µM at 48 hr incubation (Suppl Fig. 1A). Moreover, vital staining (Trypan blue) performed on BEV, RES (100 μM) and BEV + RES treated cells and untreated controls did not show any difference in the percentage of cell viability (Suppl Fig. 1B). MTT assay performed on treated and untreated ARPE-19 cells also did not show any difference in the percentage of cell viability (Suppl Fig. 1C). There are several studies reporting no toxicity on treating ARPE-19 cells with 100 μM of RES and incubation for 48 hrs^31,35,61^. Hence, we used 100 μM of RES in combination with a clinical concentration (0.25 mg/ml) of BEV in ARPE-19 cells for the study. Furthermore, we investigated the cellular apoptosis levels by looking for activated caspase levels by FLICA immunofluorescence staining. There was no difference in the FLICA positivity between the treated and untreated cultures.(Suppl Fig. 2). In the presence of RES there was a significant down regulation (p = 0.002) of the mRNA levels of vegf r2 compared to controls. A similar significant decrease (p = 0.005) in the mRNA levels of cells treated with BEV + RES was also noted compared to the controls. It has been shown that RES downregulates VEGFR2 phosphorylation and its further activation in cultured endothelial cells^62^. Apart from mRNA expression, VEGF A as well as VEGF R2 are significantly decreased in comparison to the controls. The anti-angiogenic properties of RES are mitigated via the VEGF R2. There was no significant decrease in the mRNA levels of vegf r1 in treated cells and it remained unaffected in comparison to the untreated cells. There are others studies which have also shown that VEGF-R1 remains unaffected by RES^63^.

There was a significant difference in the mRNA levels of VEGF A between the controls and RES (p = 0.043) as well as RES + BEV (0.005) treated cells. Nagineni *et al*., have shown that RES reduces VEGF A levels most likely via the mRNA levels of vegf a^64^. Using CoCl2, Zhang *et al*., have shown that RES can reduce VEGF A secretion levels similar to untreated controls^62^. In our experiments, BEV completely blocked the secreted VEGF A whereas RES reduced VEGF A levels by approximately 50%. Additionally, immunofluorescence for VEGF A revealed a decrease (50%) of intracellular VEGF A levels in cultured RPE cells in the RES treated cells compared to control cells. The decrease in the mRNA levels of VEGF A followed by a decrease in the intracellular and secreted VEGF A levels in the RES treated cultures suggests that RES is probably acting through the regulation of mRNA levels. Alex *et al*., using a zebrafish model, have already shown that the effect of RES on secreted VEGF A is through the regulation of mRNA levels^63^. Seong *et al*., have also shown in ARPE-19 cells cultured in hypoxia conditions that RES suppressed secreted VEGF levels by suppressing the chemokine receptor CXCR4^65^. It has been shown that BEV reduces intracellular and secreted VEGF A levels in comparison to controls^7,66^. The reduction of intracellular VEGF A probably due to the accumulation of BEV in the intracellular vesicles, thereby blocking the intracellular pool of VEGF A^16^. A decrease in the secretory VEGF A is secondary to the binding of BEV to the secreted VEGF A^16^. RES on the other hand, probably works on at the transcriptional level of vegf a, as it regulates the vegf a mRNA. Hence, in a combination of BEV and RES, though BEV blocks the VEGF A levels by intracellular accumulation, RES overcomes the blockage by mediating the transcriptional level of vegf a, thereby partially restoring the levels.

Zhang *et al*., revealed that a long term incubation with BEV induces EMT in cultured RPE cells by activating Notch signaling^52^. Whereas, Subramani *et al*., showed that a short pulse of BEV blocked Notch signaling activation and induced ctgf mRNA levels, thereby inducing EMT transition in cultured RPE cells^7^. Ishikawa *et al*., have shown that RES induces MET (mesenchymal-epithelial transition) in cultured RPE cells by inhibiting TGF-β induced EMT by deacetylating SMAD4^31^. Chen *et al*., using TGF-β induced EMT in ARPE-19 cells showed that RES suppressed the phosphorylation of SMAD2 and SMAD3 thereby modulated the EMT^33^. Bonyadi Rad *et al*., have shown using melanoma cells, that activated Notch 4 repressed expression of Vimentin leading mesenchymal-epithelial transition (MET)^67^. Previously, we have shown that BEV represses Notch 4 on a short incubation and induces EMT in cultured RPE cells^7^. In the present study we show that RES activates Notch 4 and reduces expression of VIMENTIN. Hence, most likely RPE cells treated with RES and BEV induce MET by the activation of Notch 4. King *et al*., have shown that RES inhibited proliferation of cultured RPE cells by blocking activation of Erk 1/2 and MEK in the presence and absence of H2O2^68^.

Our data shows that in the presence of RES, there is a significant decrease in the proliferation of cultured RPE cells as shown by BrdU, Ki67 and CYCLIN D1 positivity. Western blot and ELISA analysis show that there is decrease in phosphorylated Erk and MEK in cells treated with RES. This finding are similar to what has been shown by other’s wherein the dampening of RPE proliferation has been attributed to be via Erk and MEK pathways. We as well as others have shown previously that phagocytosis of RPE cells is reduced in the presence of BEV treatment^7,13,14^. It has been shown that inhibition of autophagy associated with reduced phagocytic activity in ARPE-19 cells treated with 3-methyladenine^69^. We were further interested on assessing the levels of LC3 and p62 in ARPE cells treated with or without BEV, RES respectively. Here we show that in the presence of BEV with RES, the phagocytic property of RPE is restored to the controls levels. ARPE-19 cells treated with BEV, showed increase in the LC3-II and p62 levels compared to untreated or controls suggesting that blockage of autophagy degradation. Conversely, cells treated with BEV and RES showed decreased levels of LC3-II and p62. There could be a possibility that RES mediated induction of autophagy enhances the autophagic lysosomal clearance in ARPE cells treated with BEV^34^. Ferguson *et al*., have shown a convergence of phagocytosis with autophagy, wherein there is conversion of LC3 I subunit to II, enabling a proper degradation of phagocytosed photoreceptor outer segments^70^. Though our results provide a clue of a possible interaction of phagocytosis and autophagy, further studies are needed to investigate in depth the signaling crossover of phagocytosis and autophagy using pharmacological activators and blockers of autophagy. With the expression of LC3 protein regulation, it can be well envisaged that the phagocytosis of RPE is LC3-associated phagocytosis with involvement of non-canonical autophagy^71^. Grandbarbe *et al*., have shown that activated Notch signaling can induce phagocytosis in microglia cell^72^. Activated Notch signaling has been shown to activate autophagy in gliobalstoma cells^73^.

The transmembrane potential is readout of the blood-retinal barrier function and its integrity^7,74^. We had previously shown that a short exposure to BEV induced RPE membrane depolarization^7^. Our present experiments showed that there was a significant decrease in the depolarization of transmembrane potential in the presence of RES when compared to cells incubated with BEV alone. This suggests a protective role of RES in restoring the membrane integrity of blood-retinal barrier in the presence of BEV. Notch signaling is highly conserved developmentally regulated signaling pathway^47^. We previously reported that in a short exposure of BEV, NOTCH 4 and DLL 4 were blocked. Pinchot *et al*., using a high throughput screening found that Notch signaling is activated by RES^45^. Gidfar *et al*., have shown that RES can activate Notch signaling in meibomian gland epithelial cells^46^. Here, we show that in the presence of RES, Notch signaling gets activated. This activation along with dephosphorylation of Erk 1/2 and MEK has been shown to be major drivers for the functional restoration of RPE cells treated with RES + BEV compared to those treated with BEV alone.

Further studies using *in–vitro* diseased models such as culturing the cells under oxidative stress of hypoxia/hyperoxia in the presence and absence of RES would provide insight into its clinical application. Additional work using animal disease models can reconfirm our findings and may provide a tool in the future to pave way for a combinatorial therapy for ocular neo-vascular diseases.

The advantage of RES is primarily its non-toxic nature. It is FDA approved and already a part of several clinical trials (NCT02625376, NCT02321189, NCT02321176). Our results provide an impetus to further investigate the beneficial role of RES in combination with BEV for patients with neo-vascular diseases. This study was done with a short term single pulse of BEV. It would be of interest to determine the role of RES in reversing the adverse effects of BEV on long-term repeated usage. Experiments with animal models could provide evidence of additional secondary effects of combination treatment with BEV + RES. Our findings can pave way for a newer treatment strategy for patients with AMD and PDR that can reduce the complications of anti-VEGF treatment regime. Partial reversal of the adverse effects of BEV by RES suggests an additional role of RES beyond its anti-oxidative properties.

## Materials and Methods

### Cell culture and reagents

The human retinal pigment epithelial cell line (ARPE-19) was provided by Dr Anders Helder, Karolinska Institute, Sweden. Cells were maintained in 5.5 mM/L glucose Dulbecco’s modified Eagle’s medium (Himedia, Mumbai, India) supplemented with 10% fetal bovine serum (Gibco, Life Technologies, Carlsbad, USA) in a humidified atmosphere of 5%CO2 at 37 °C. Cells were seeded and grown to a sub-confluence of 60–70% in normoxia.

### BEV and RES treatment

ARPE-19 cells were seeded onto 12-well tissue culture plates at a density of 2 × 10^5^ cells per well. Semi confluent cultures (60–70% confluence) were serum starved for 24 hours before the treatments. Treatment strategy for the three groups of experiments was as follows:

BEV group - ARPE-19 cells were treated with a clinical concentration (0.25 mg/ml) of BEV (Roche Diagnostics, GmbH, Germany). After two hours of BEV treatment the media was collected and replaced with fresh media (with no BEV) and followed for 46 hours with a total conditioned treatment of 48 hours.

RES group - ARPE-19 cells were treated with 100 µM of RES (Sigma, St.Louis, MO, USA) for 48 hours.

BEV + RES group - ARPE-19 cells were treated with BEV for 2 hours and then replaced by RES for 46 hours.

All untreated cells served as controls. The supernatants and the cells of treatment and controls groups were collected for the measurement of VEGF levels by sandwich ELISA (R&D systems™, Minneapolis, USA) and other molecular/biochemical analysis.Experiments were carried out in triplicate and ARPE-19 cells with a passage number of 9–12 were used for this study.

### Cell Viability count - Trypan Blue

Cultured ARPE-19 cells treated with BEV, RES, BEV + RES, as well as the control cells were stained with supravital, Trypan blue. The procedure for trypan blue is well documented; cells are diluted to a suitable concentration and 0.4% (w/v) trypan blue (Mediatech, Cellgro, USA) is added to the media containing cells^75^. Then viable and non-viable cells were counted manually using a haemocytometer and the percentages of viable cells were ascertained and represented graphically.

### MTT Assay

Cultured ARPE-19 cells after 48 hrs of treatment with BEV, RES and BEV + RES, as well as the controls were assayed for metabolic activity. The colorimetric assay of (3-(4, 5-Dimethylthiazol-2-yl)-2, 5-Diphenyltetrazolium Bromide) (MTT) was performed using the standard protocol as per the manufacturer’s instruction (Xpert^TM^ MTT Cell Assay Teaching Kit, Himedia, Mumbai, India). The optical absorbance of the solution at 570 nm was measured using microplate reader 680 (Bio-Rad™, California, USA). The experiment was executed in triplicates and represented graphically as a percentage of the viability.

### Caspase activity (FLICA)

Caspase activity was carried out on cultured ARPE-19 cells treated with BEV, RES and BEV + RES as mentioned earlier^76^. In brief, 48 hours after treatments, active caspase staining was performed using a fluorochrome inhibitor of caspases (FLICA) kit (Imgenex, San Diego, CA, USA) per the manufacturer’s instructions. Fluorescence staining was documented using the ProgRes Capture Pro 2.5 software on an Olympus BX41 fluorescent microscope (Olympus, Shinjuku, Tokyo, Japan). The fluorescence intensity was quantified using Image J 1.48 version software (http://imagej.nih.gov/ij/; provided in the public domain by the National Institutes of Health, Bethesda, MD, USA) and represented graphically.

### Quantitative real-time (qRT) PCR

Total cellular RNA was extracted using the TRIzol® reagent (Ambion, Carlsbad California, USA) from cells of each group as mentioned previously^77^. Briefly, equal amounts of extracted RNA were converted to cDNA using the High-Capacity cDNA Reverse Transcriptase Kit (Life Technologies, Carlsbad, USA) according to the manufacturer’s instructions and stored at −20 °C. Quantitative RT-PCR was performed on the Bio-Rad CFX Connect TM (Bio-Rad, California, USA) sequence detector using the SYBR® Green PCR Master Mix (Life Technologies, Foster City, USA). The results were analyzed according to manufacturer’s instructions. The list of gene specific primers used is provided in Table 1.

**Table 1:** Primers used for quantitative real-time PCR.

S. No	Gene name	Sequences(5′–3′)	Product size	Gene acc no
1	gapdh	FP: ACCCACTCCTCCACCTTTGAC	100	NM_001289746.1
		RP: TGTTGCTGTAGCCAAATTCGTT		
2	vegf a	FP: TGCAGATTATGCGGATCAAACC	81	NM_001287044.1
		RP: TGCATTCACATTTGTTGTGCTGTAG		
3	vegf r1	FP: CAGGCCCAGTTTCTGCCATT	82	NM_002019.4
		RP: TTCCAGCTCAGCGTGGTCGTA		
4	vegf r2	FP: CCAGCAAAAGCAGGGAGTCTGT	87	NM_002253.2
		RP: TGTCTGTGTCATCGGAGTGATATCC		
5	collagen I (extracellular matrix protein)	FP: TTGTGCGATGACGTGATCTGT	111	NM_000088.3
		RP: TTGGTCGGTGGGTGACTCTG		
6	collagen IV (extracellular matrix protein)	FP: GCAAACGCTTACAGCTTTTGG	69	NM_001845.5
		RP: GGACGGCGTAGGCTTCTTG		
7	Fibronectin (mesenchymal marker)	FP: TGGCCAGTCCTACAACCAGTA	119	NM_001306129.1
		RP: CTCGGGAATCTTCTCTGTCAGC		
8	α – sma (mesenchymal marker)	FP: GCTGGCATCCATGAAACCAC	104	NM_001613.2
		RP: TACATAGTGGTGCCCCCTGA		
9	Vimentin (mesenchymal marker)	FP: ACACCCTGCAATCTTTCAGACA	76	NM_003380.3
		RP: GATTCCACTTTGCGTTCAAGGT		
10	e-cadherin (epithelial marker)	FP: AAGGAGGCGGAGAAGAGGAC	87	NM_004360.3
		RP: CGTCGTTACGAGTCACTTCAGG		
11	pcna	FP: GCCAGAGCTCTTCCCTTACG	87	NM_002592
		RP: TAGCTGGTTTCGGCTTCAGG		
12	ki67	FP: CTTTGGGTGCGACTTGACG	199	NM_002417
		RP: GTCGACCCCGCTCCTTTT		
13	cdc20	FP: GTTCGGGTAGCAGAACACCA	187	NM_001255
		RP: CCCCTTGATGCTGGGTGAAT		
14	cyclin d1	FP: AGCTCCTGTGCTGCGAAGTGGAAAC	133	NM_053056
		RP: AGTGTTCAATGAAATCGTGCGGGGT		
15	mertk	FP: GTGCAGCGTTCAGACAATGG	83	NM_006343.2
		RP: TCGATGTAGATGGGATCAGACAC		
16	notch4	FP: GAGGACAGCATTGGTCTCAAGG	61	NM_004557.3
		RP: CAACTCCATCCTCATCAACTTCTG		
17	dll4	FP: AAGGCTGCGCTACTCTTACC	90	NM_019074.3
		RP: AAGTGGTCATTGCGCTTCTT		
18	p62	FP: GACTACGACTTGTGTAGCGTC	139	NM_001142299
		RP: AGTGTCCGTGTTTCACCTTCC		
19	lc3a	FP: CGTCCTGGACAAGACCAAGT	181	NM_032514
		RP: CTCGTCTTTCTCCTGCTCGT		
20	lc3b	FP: AGCAGCATCCAACCAAAA	187	NM_022818
		RP: CTGTGTCCGTTCACCAACAG		

### ELISA assay

Levels of secreted VEGF in the cell supernatant of controls, and treated (BEV, RES and BEV + RES) samples were estimated using the human VEGF Duoset sandwich ELISA (R&D systems™, Minneapolis, USA) as mentioned previously^78^. As per the manufacturer’s instruction, the samples and standards were run in triplicates. The reaction product was quantified using the micro plate reader 680 (Bio-Rad™, California, USA) at a wavelength of 450 nm with the reference filter at 570 nm. The quantity of phosporylated p-MEK1/2 (Ser217/221), p44/42MAPK(Thr202/Tyr204),p-SAPK/JNK(Thr183/Tyr185),p-Akt1(Ser473) and p-Akt1(Thr 308) were measured from cell lysates using human PathScan^®^ ELISA kits (Cell Signaling technology,Beverly,MA,USA). Briefly, as per the manufactures instruction, the treated and control ARPE-19 cells were lysed using cell lysis buffer followed by sonication on ice and centrifuged at 10000xg for 10 min at 4 °C. The cell lysate supernatants was used for analyzing the phosphorylated kinase proteins and quantified by absorbance at 450 nm using microplate reader 680 (Bio-Rad™, California, USA).

### BrdU assay

The cell proliferation rate was assessed as mentioned earlier using the BrdU labeling kit (BD Biosciences, San Diego CA, USA) in BEV and BEV + RES treated ARPE-19 cells^7^. Briefly, after BEV and BEV + RES treatment, the cells were incubated with BrdU and then collected and stained as per the manufacturer’s instructions. Using flow cytometry, FACS caliber™ (BD Biosciences, San Diego, CA, USA) stained cells were acquired and analyzed in the FL1 channel using the BD CellQuest™ Pro software (BD FACSCaliber™, San Diego, CA, USA).

### Immunofluorescence staining

ARPE-19 cells (1 × 10^3^) were grown on cover slips (12 mm, Blue Star, India) and treated with/without BEV, RES and BEV + RES and stained for specific markers as previously mentioned^79^. Cultured cells were fixed with 4% paraformaldehyde and permeabilized with 0.1% Triton X-100 for 10 minutes. The cells were blocked with 1% bovine serum albumin for 15 minutes and then incubated overnight with specific primary antibodies at 4 °C. On the next day, cells were stained with secondary antibodies (1:500) for 2 hours at RT. After washing, the cells were mounted with the VECTASHIELD® mounting medium and DAPI (vector laboratories, CA, USA). Fluorescence images were documented on the ProgRes Capture Pro 2.5 software on an Olympus BX41 fluorescent microscope (Olympus, Shinjuku, Tokyo, Japan). Fluorescence intensity was quantified using the Image J 1.48 version software. The list of primary and secondary antibodies is provided in Table 2.

**Table 2:** List of Primary and Secondary antibodies.

No	Name of protein	Dilution	Company
**Immunofluorescence**			
1	VEGF Rabbit	1:100	CusAb, USA
2	ZO-1 Rabbit	1:100	Boster Biologicals, CA, USA
3	Alexa Fluor 488 phalloidin	1:100	Thermo Fisher Scientific, India
4	Ki67 Rabbit	1:200	Imgenex, India
5	NOTCH4 Mouse	1:100	Cell signaling, USA
6	DLL4 Rabbit	1:100	Abcam, UK
**Western Blot**			
7	GAPDH Mouse	1:1000	Abgenex, India
8	p62 Rabbit	1:1000	Cell signaling, USA
9	LC 3 (I and II) Rabbit	1:1000	Cell signaling, USA
10	VEGF R2 Mouse	1:1000	Abgenex, India
11	VEGF Rabbit	1:1000	CusAb, USA
12	Total Erk 1/2 Rabbit	1:1000	Cell signaling, USA
13	Phospho Erk 1/2 Rabbit	1:1000	Cell signaling, USA
14	Total MEK Mouse	1:1000	Cell signaling, USA
15	Phospho MEK Rabbit	1:1000	Cell signaling, USA
16	VIMENTIN Rabbit	1:1000	Abgenex, India
17	E-CADHERIN Mouse	1:1000	Abgenex, India
18	CYCLIN D1 Mouse	1:1000	Abgenex, India
19	NOTCH4 Mouse	1:1000	Cell signaling, USA
20	DLL4 Rabbit	1:1000	Abcam, UK
21	HES1 Mouse	1:1000	EnoGene, USA
**Secondary Antibodies**			
22	Goat anti-mouse IgG, HRP	1:4000	Biolegend, USA
23	Donkey anti-Rabbit IgG, HRP	1:4000	Biolegend, USA
24	Goat anti-mouse IgG, Alexa Flour 488	1:500	Abgenex, India
25	Donkey anti-Rabbit IgG, Cy3	1:500	Jackson Immuno
26	Donkey anti-Rabbit IgG, DyLight 488	1:500	Biolegend, USA

### Phagocytosis assay

Cultured ARPE-19 cells were treated with BEV, RES and BEV + RES or left untreated as described previously^7^. Briefly, the cells were first incubated with FITC labeled latex beads for 48 hours (1:50, Cayman Chemicals, Ann Arbor, Michigan, USA) as per the manufacturer’s instructions. The cells were then trypsinized and analyzed in the FL1 channel by FACS caliber (BD Biosciences, San Diego, CA, USA). For immunofluorescence, the cells were cultured on coverslips and incubated with latex beads post treatments for 48 hours at 37 °C. The coverslips were mounted with the VECTASHIELD® mounting medium and DAPI (Vector laboratories, Burlingame, California, USA) and observed under a fluorescence microscope (Carl Zeiss Oberkochen, Germany). The number of DAPI positive cells with latex beads near or around the nucleus were counted manually and represented graphically.

### Western blot analysis for protein expression

For total protein extraction, cells were lysed with the RIPA lysis buffer (25 mM Tris, 150 mM Sodium Chloride, 1% NP-40, 1% Sodium Deoxycholate, 0.1% SDS; G- Bioscience, St. Louis, MO, USA) with a protease inhibitor cocktail. The cell lysates were collected after centrifugation and mixed with 5X SDS sample buffer. The samples were loaded and separated on 10% SDS-PAGE, and then wet-transferred using PVDF membranes (Rugby WAR, UK). The membranes were blocked in 5% skimmed milk powder in TBS-T and incubated overnight with specific primary antibodies at 4 °C. After washing with PBST, the membranes were incubated with HRP conjugated secondary antibodies for 1 hour at RT. The protein bands were visualized using a chemiluminescence detection kit (Pierce® ECL Plus, Thermo scientific, Rockford, IL, USA) using with the image Quant LAS 500 gel documentation/chemiluminescence detector (GE Healthcare Life Science, Uppsala, Sweden). The list of primary antibodies and secondary antibodies used for western blot are provided in Table 2.

### Transmembrane potential assay

Membrane potential assay was carried out on cultured ARPE-19 cells treated with BEV, RES and BEV + RES and the analysis were done as mentioned earlier^7^. In brief, after 48 hours of various treatments, the cells were trypsinized and incubated with 20 nM of Bis- (1, 3-Dibutylbarbituric Acid) Trimethine Oxonol [DiBAC4(3)] for 30 mins at 37 °C. The intensity of the dye intake was analyzed in flow cytometry in the FL1 channel using the BD CellQuestTM Pro software.

### Scratch assay

ARPE-19 cultures were grown to confluence in 35 mm tissue culture dishes. Using a sterile 1000 μl tip, cultures were scraped in a straight line to create a scratch. Care was taken to create scratches of similar width in the control as well as treatment groups. Cultures were washed with 1XPBS twice to remove detached and floating cells and cellular debris. The cells were then treated with BEV, RES and BEV + RES, wherein BEV treatment was given for 2 hours followed by RES treatment for 22 hours. The treated culture dishes were placed in the tissue culture incubator at 5% CO_2_ and 37 °C. After 24 hours, the cells were observed using a phase contrast microscope and photographed with referenced matching points. In the images acquired, the distance between the scratches were measured using the Image J 1.48 version software (NIH, Bethesda, MD, USA).

### Statistical analysis

All experiments were performed in triplicate and results of three independent experiments were used for statistical analysis. Data were represented as the mean ± SD and were analyzed with the Student’s t-test. Student’s t-test was calculated between control sample and BEV treated, BEV and BEV + RES treated samples. Significance value denoted, p* < 0.05, ** < 0.01, *** < 0.005. The specific p-value is mentioned when the p ≥ 1 × 10^–4^, and when the p ≤ 1 × 10^–4^, it is stated as p ≤ 0.005.

### Data Availability

All data generated or analysed during this study are included in this published article (and its Supplementary Information files).

## Electronic supplementary material

Supplementary Information
