# Supplementary material for: Resveratrol reverses the adverse effects of bevacizumab on cultured ARPE-19 cells
Source: Sci Rep. 2017 Sep 25;7:12242. doi: 10.1038/s41598-017-12496-z (PMC5612947; doi:10.1038/s41598-017-12496-z)
Supplement: Supplementary file 1 — Supplementary Information [file 41598_2017_12496_MOESM1_ESM.pdf]

Resveratrol reverses the adverse effects of bevacizumab on cultured ARPE-19 cells

Murali Subramani[1,5], Murugeswari Ponnulagu[1], Lekshmi Krishna[1], Nallathambi Jeyabalan[2], Priyanka Chevour[2], Anupam Sharma[2], Chaitra Jayadev[3], Rohit Shetty[4], Nargis Begum[5], Govindaraju Archunan[6], Debashish Das[1]*

1 Stem Cell Lab, GROW Laboratories, Narayana Nethralaya Foundation, Bangalore, Karnataka, India

2 GROW Laboratories, Narayana Nethralaya Foundation, Bangalore, Karnataka, India

3 Vitreoretina Services, Narayana Nethralaya Eye Hospital, Bangalore, Karnataka, India

4 Department of Cornea and Refractive Surgery, Narayana Nethralaya Eye Hospital, Bangalore, Karnataka, India

5 Postgraduate Department of Biotechnology, Jamal Mohammed College, Tiruchirappalli, Tamilnadu, India

6 Department of Animal Science, Bharatidasan University, Tiruchirappalli, Tamilnadu, India

*Corresponding address:

Dr Debashish Das

Stem Cell Research Laboratory, GROW Laboratory,

Narayana Nethralaya Foundation, Narayana Nethralaya Eye Institute,

Narayana Health City, Bommasandra, Bangalore- 560 099, Karnataka, India

Fax No: +91 80 6666 0650

Tel No: +91 80 6666 0722

Email: drdebashish@narayananethralaya.com;

dasdebashish@yahoo.co.uk

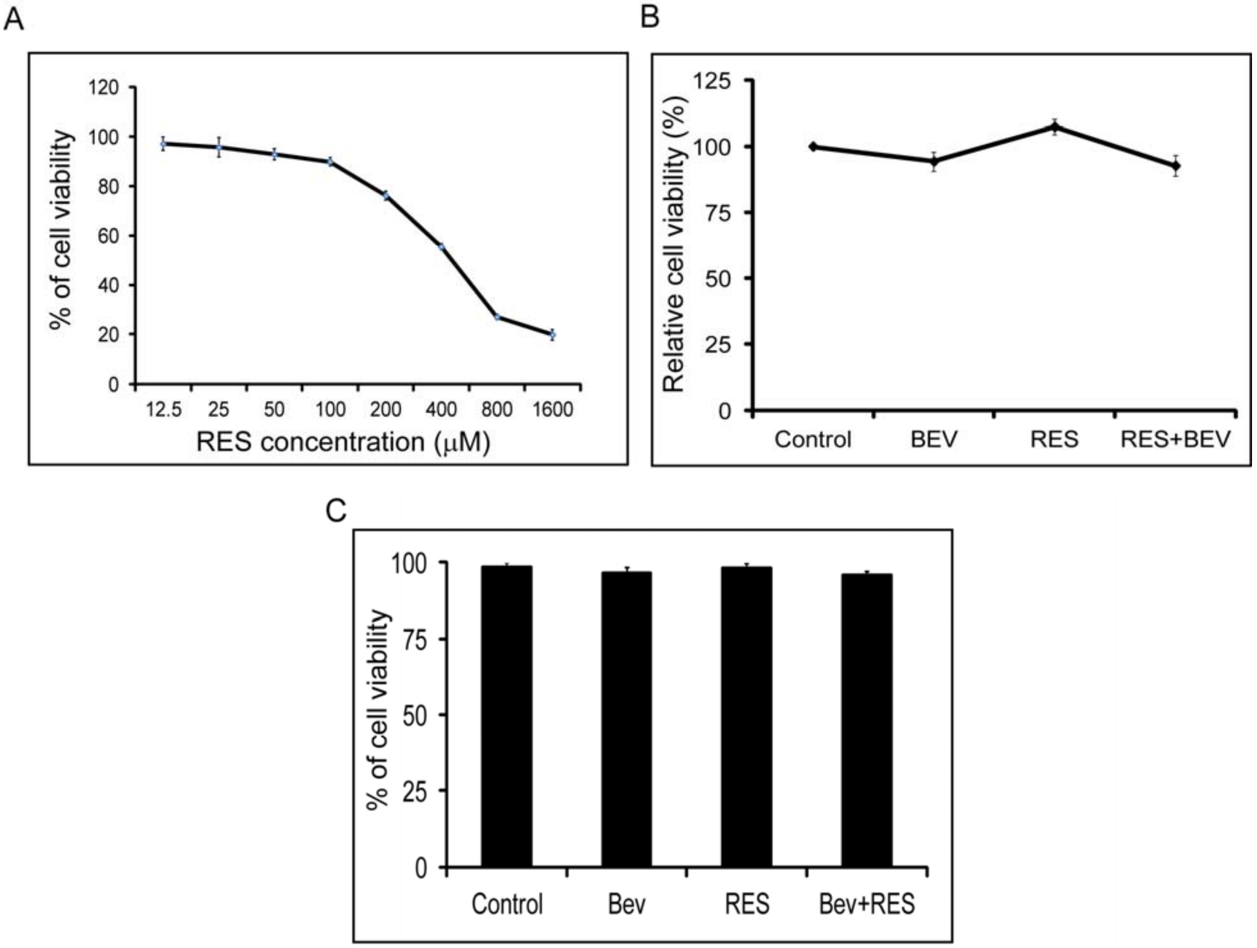

Supplementary Figure 1:

RES toxicity and IC50 assay on ARPE-19 cells

ARPE-19 cells were incubated with different concentrations of RES with increasing dosage for 48hrs. The cells were then proceeding for MTT assay to determine the IC50 dosage for ARPE-19 cells. The percentage of cell viability is plotted and the results revealed that the IC50 for RES on cultured ARPE-19 is 625.3μM (A). The results also show that the toxicity of RES at 100μM is similar to the untreated controls. Hence, for all the further experiments we have treated the cells with 100μM. MTT assay on the ARPE-19 cells treated with BEV, RES or both were similar to the untreated control levels (B). Trypan blue dye exclusion analysis for determining the number of live cell was done in triplicate. The results show that there was no toxicity related cell death induced by 100μM of RES incubated for 48hr in cultured ARPE-19 cells (C)

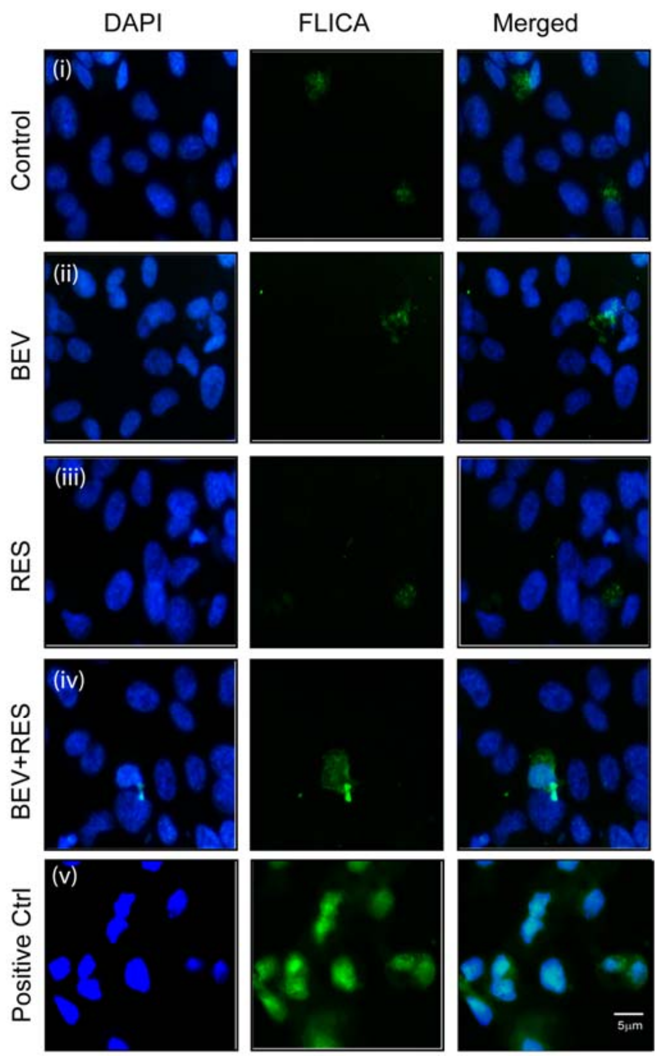

Supplementary Figure 2:

ARPE-19 apoptosis levels in the presence of BEV+RES

ARPE-19 cells were cultured in the presence of BEV, RES and BEV+RES or left untreated for 48hrs. FLICA staining was performed on the treated and untreated cells to determine the active caspase staining (i-iv). UV-A exposed ARPE-19 cells were used as positive control for FLICA staining (v). Representative FLICA stained immunofluorescence images have been depicted. Scale bar = 5µm.
